# Acceptability and feasibility of an adapted Portuguese folk dance–based therapy in older adults with moderate-to-severe dementia and balance impairment

**DOI:** 10.3389/fnagi.2026.1886478

**Published:** 2026-08-20

**Authors:** Ana Almeida, Vasco Caveirinha, Joana Felício, Rui Fontes, Patrícia João, Tânia Libório, Ana Piedade, Carlos Família, Catarina Godinho, Júlio Belo Fernandes

**Affiliations:** 1 Residência Sénior Egas Moniz, Sesimbra, Portugal; 2 Egas Moniz Center for Interdisciplinary Research (CiiEM), Egas Moniz School of Health & Science, Caparica, Portugal; 3 Forensic and Psychology Sciences Laboratory Egas Moniz, Campus Universitário Quinta da Granja, Caparica, Portugal; 4 Nurs* Lab, Almada, Portugal

**Keywords:** acceptability, dementia, feasibility, long-term care, older adults, physiotherapy, Portuguese folk dance, rehabilitation

## Abstract

**Introduction:**

Population ageing has increased the prevalence of dementia, often accompanied by mobility impairments. It is essential to develop effective interventions and promote adherence among people with dementia. Culturally meaningful activities may enhance motivation, engagement, and rehabilitation outcomes.

**Methods:**

A mixed-methods pilot study was developed to evaluate the acceptability and feasibility of an adapted Portuguese folk dance–based intervention among older adults with moderate to severe dementia and balance impairment in a long-term care facility.

**Results:**

Ten participants were recruited; eight completed the 4-week, twice-weekly programme. Quantitative measures included the Mini-BESTest and MoCA. Acceptability domains, including enrolment, retention, attendance, satisfaction, and safety, were assessed through surveys and interviews. Retention was 80%, and attendance averaged 85.9%. No adverse events occurred. All participants expressed high satisfaction, perceived the programme as useful, and indicated a willingness to repeat or recommend it. Balance improved significantly [Mini-BESTest mean change = 1.38 ± 0.32; *t* = 4.25, *p* = 0.004, Cohen’s *d* = 1.50; BF_10_ = 14.00 (numerical error ≈ 5.76 × 10^−7^)], while cognition remained stable [MoCA *w* = 0.00, *p* = 1.00; BF_10_ = 0.50 (numerical error 9.03 × 10^−5^)]. Qualitative data highlighted enjoyment, motivation, and social connection as facilitators of adherence. The adapted Portuguese folk dance–based programme showed feasibility and acceptability among older adults with dementia.

**Discussion:**

The findings support its feasibility and preliminary potential as a culturally rooted intervention, warranting evaluation of balance-related benefits in future controlled trials.

## 1 Introduction

Population ageing has become one of the defining demographic trends of the 21st century (United Nations Department of Economic and Social Affairs, Population Division, 2023; World Health Organisation, 2025a). As life expectancy increases across most regions, the prevalence of neurodegenerative diseases, particularly dementia, has risen substantially (Wang et al., 2024). Dementia is characterised by a progressive decline in memory, executive function, and communication, ultimately leading to dependence in daily activities (World Health Organisation, 2025b). Beyond cognitive deficits, people living with dementia frequently exhibit deterioration in motor function, postural control, and balance (Andrade-Guerrero et al., 2024; Fujita et al., 2024). As a result, they are more likely to experience falls, fear of movement, and loss of confidence, particularly in certain subtypes such as dementia with Lewy bodies, which further restricts physical activity and social interaction (Kim and Kim, 2024; Soysal et al., 2021). Taken together, these impairments have a profound impact on quality of life, social participation, and caregiver burden (Jayakody and Arambepola, 2022).

With the growing number of individuals affected, prioritising interventions that maintain mobility and promote meaningful engagement is essential to support healthy ageing and quality of life. Physical activity and exercise are increasingly recognised as components of dementia management. In people with moderate dementia, exercise may help maintain functional ability and cognition, although this recommendation is based on low to very low certainty evidence (Veronese et al., 2023). Guidance for people with more advanced impairment is correspondingly limited. Exercise programmes targeting balance, strength, and mobility are also relevant components of fall prevention and functional rehabilitation in people with cognitive impairment (Lam et al., 2018b; Montero-Odasso et al., 2022). However, conventional exercise programmes often have limited adherence among individuals with dementia (Di Lorito et al., 2020).

People with moderate-to-severe dementia present distinct practical challenges for exercise-based interventions. Difficulties in sustaining attention, understanding and retaining instructions, sequencing movements, communicating needs, and maintaining postural control may limit participation in multistep activities and increase the need for closer supervision and ongoing adaptation. This population also remains underrepresented in exercise research. For example, the PrAISED programme recruited people with mild cognitive impairment or early dementia and MoCA scores of 13–25, whereas the ENJOY trial excluded people with severe dementia and those unable to stand or walk without physical assistance (Bajwa et al., 2019; Levinger et al., 2023). Moreover, a systematic review focused specifically on exercise interventions for people with moderate-to-severe dementia identified only eight eligible studies and concluded that the available evidence was of low quality (Long et al., 2019). Feasibility, safety, and acceptability therefore need to be established directly in this population rather than inferred from studies conducted at earlier stages of cognitive impairment.

These challenges have practical implications for intervention design. People with more advanced dementia may be better able to engage in activities that minimise reliance on complex verbal instructions and instead use clear demonstration, repetition, additional processing time, and individualised physical support. Responsiveness to music may remain relatively preserved even in later or severe stages of dementia, providing a further rationale for incorporating familiar music and rhythm into appropriately adapted interventions (Baird and Samson, 2015). Cognitive and physical accessibility alone, however, may not be sufficient to sustain participation. There is growing recognition that therapeutic activities must be culturally grounded and meaningful, reflecting the person’s identity, values, and life story (Asnaani and Hofmann, 2012; Conn et al., 2013). Meaningful engagement fosters motivation, emotional connection, and sustained participation, key elements for effective care for people with dementia (National Institute for Health and Care Care Excellence, 2018; Smebye and Kirkevold, 2013).

Culturally adapted interventions have gained attention as an avenue to enhance both acceptability and therapeutic effectiveness. In Portugal, traditional music and dance are deeply embedded in the nation’s heritage and collective memory. Portuguese folk dance, performed in community ensembles known as *ranchos folclóricos*, originated in rural settings and represents a dynamic expression of local identity, social cohesion, and joy. It integrates rhythmic movement with regional songs, typically performed in groups where participants coordinate circular and linear sequences of steps. As documented in the Portuguese ethnographic literature, despite regional variations, these dances share key characteristics: rotational patterns, rhythmic footwork based on two- or three-step combinations, and group formations that promote synchrony and cooperation (Ribas, 1983; Ribeiro et al., 2020). Beyond their artistic value, these features offer therapeutic potential by stimulating coordination, balance, and social interaction in a context that feels familiar and emotionally rewarding for many older Portuguese adults (Fernandes et al., 2023).

Given that the combination of complex and high-impact movements characteristic of tango and Latin dance has been shown to improve coordination and balance in individuals with dementia (Aguiñaga and Marquez, 2017; Bracco et al., 2023), we hypothesise that a culturally adapted Portuguese folk dance may provide similar benefits when implemented with appropriate safety measures and cognitive adaptations.

Dance-based therapies have increasingly been explored as multimodal interventions that integrate physical, cognitive, emotional, and social dimensions of rehabilitation. Recent systematic reviews suggest that dance interventions may support balance, gait, muscle strength, functionality, and cognitive function among older adults with cognitive impairment and dementia (Menezes et al., 2022; Tao et al., 2023). Dance interventions may also contribute to psychological wellbeing and social engagement among older adults more broadly (Fonseca et al., 2025). Evidence from Latin dance and tango interventions is also encouraging, although Latin dance has primarily been examined among people with mild cognitive impairment (Aguiñaga and Marquez, 2017; Bracco et al., 2023). However, the evidence remains heterogeneous, and relatively few studies have examined dance interventions among people with dementia living in residential care (Manji et al., 2024).

More broadly, most published research has focused on general dance movement therapy or established international dance styles. Dance forms that incorporate culturally significant music and community-oriented movement patterns, such as Portuguese folk dance, remain underexplored. To our knowledge, no previous study has evaluated the therapeutic use of an adapted Portuguese folk dance intervention for people with dementia. Addressing this gap is relevant not only for advancing culturally responsive rehabilitation practices but also for expanding non-pharmacological strategies that promote autonomy and wellbeing in dementia care. Therefore, the present study aims to evaluate the acceptability and feasibility of an adapted Portuguese folk dance–based therapy for older adults with moderate-to-severe dementia and balance impairment. By assessing participants’ engagement, safety, and preliminary functional outcomes, this work seeks to establish the foundations for future controlled trials examining its clinical efficacy.

## 2 Methods

### 2.1 Study design

This study employed a mixed-methods design, integrating both quantitative and qualitative approaches, structured into two distinct yet sequential and complementary phases.

### 2.2 Study setting

The study was carried out in a long-term care facility for older adults in Portugal. The facility offers 24-h support, including nursing care, basic medical monitoring, physiotherapy sessions, assistance with activities of daily living, and social and recreational activities. Residents commonly have multimorbidity and different levels of cognitive impairment and mobility issues.

### 2.3 Ethical consideration

The current study was approved by the Egas Moniz Institutional Ethics Review Board (ID: 1582). Written informed consent was obtained from participants capable of providing it, or from their legal guardians where applicable. For individuals with dementia, consent was additionally obtained from the participants themselves, alongside that of their legal guardians, to affirm their voluntary participation.

### 2.4 Sampling and recruitment

A public announcement invited every resident to register their interest in the study. All registrants then underwent a screening to determine eligibility. During the initial phase, two nurses supervised the recruitment process utilising information from patients’ clinical records, including diagnoses, disease stages, and other relevant clinical data, while adhering to the following criteria:

Inclusion criteria:

- Documented clinical diagnosis of dementia in the medical record.
- Moderate to severe dementia defined as a MoCA score of 17 or less.
- Ability to follow three-step commands.
- Ability to tolerate at least 45 min of light to moderate exercise.
- Ability to communicate with the investigator and understand and comply with study procedures (with caregiver support if needed).
- Familiarity with Portuguese folk dance, specifically Vira, and willingness to participate in the intervention.

Exclusion criteria:

- Pre-existing neurological, cardiovascular, orthopaedic, or rheumatic conditions that could interfere with safe participation in the protocol.
- Severe visual or hearing impairment not correctable with aids.
- Poor tolerance of group settings or significant behavioural disturbances likely to hinder participation or compromise safety.

Familiarity with Portuguese folk dance was assessed following the public announcement through a brief discussion with each resident, and where appropriate, a family member or caregiver. Participants were considered eligible when they were familiar with the Vira and expressed willingness to take part in the intervention. This criterion was intended to ensure that the activity was personally meaningful and that participation was voluntary; it did not require participants to prefer the Vira over other forms of exercise or activity.

In the second phase, a member of the research team supplied both oral and written information regarding the study and recommended that each prospective participant, along with their family, caregiver, or legal guardian, take at least 24 h to consider and discuss before reaching a decision.

**Figure 1:**
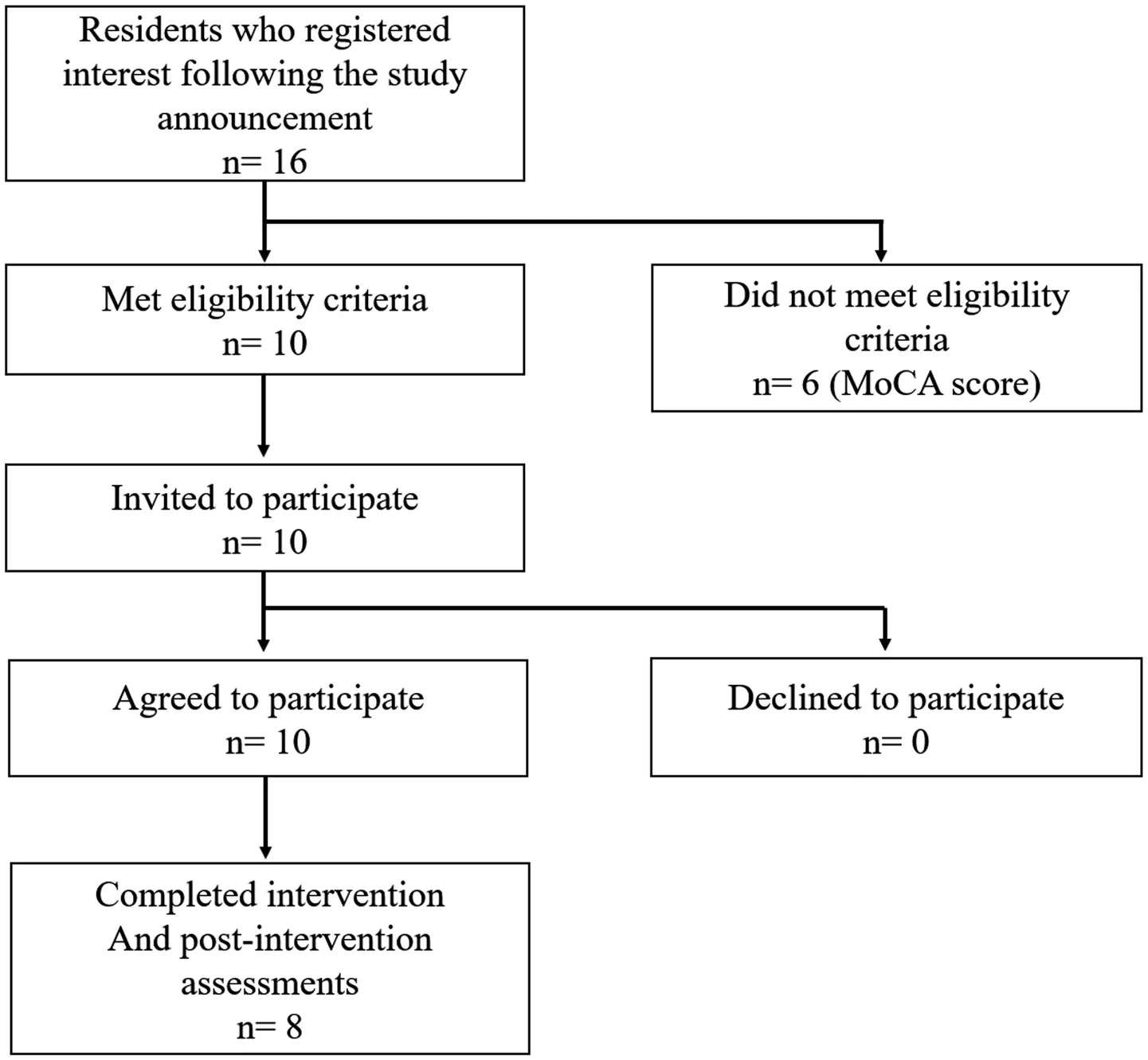
Participant flow through registration, screening, and the four-week intervention.

Participant flow through registration and screening is presented in Figure 1.

### 2.5 Intervention

The intervention comprised a four-week group programme, conducted twice a week, with each session lasting approximately 40 min. This programme was implemented as a supplement to the standard care provided at the facility, which includes daily sessions of occupational and physical therapy lasting 45 min each.

The dance sequence employed in this intervention was an adaptation of the Portuguese folk dance Vira, devised by Fernandes et al. (2023). Vira is a traditional style extensively practised throughout Portugal and carries significant cultural and social importance. The term Vira, which literally means “to turn” or “to twist”, reflects its characteristic rotational and circular movements. Typically, dancers perform in pairs, facing each other with their arms raised but without holding hands, as the group moves in a counterclockwise circle. At specific musical cues, a subset of participants steps into the centre, taps the right foot, and then reverts to their original position, after which the rotation continues, alternating the entrants into the centre with each repetition (Ribas, 1983; Ribeiro et al., 2020). Vira was chosen because it incorporates full-body movement (weight transfer, directional changes), challenges postural control and flexibility, and engages upper-limb function as well as perceptual–cognitive skills (attention, sequencing, memory).

Sessions were prioritised to ensure safe execution, incorporating explicit cues related to alignment, balance, and coordination. The progression was systematically graded, enabling participants to enhance strength, mobility, and control progressively over time. To accommodate individual requirements, partner work was excluded from this protocol. Participation was determined through a prior assessment conducted by the physiotherapist, taking into account each participant’s ability to maintain a standing position without support. Participants who were able to remain standing safely without physical support performed the intervention independently. Participants who were unable to maintain standing without support, but could participate safely with physical assistance, performed the intervention with hand contact for stability and, when necessary, a gait belt. Participants with more substantial balance impairment, for whom standing participation was not considered safe, performed the routine while seated in a wheelchair. While seated, participants performed upper- and lower-limb movements, forward and backward wheelchair propulsion, and trunk flexion, extension, and lateral flexion.

To accommodate differences in cognitive functioning, instructions were delivered one step at a time and accompanied by simultaneous demonstration by a member of the intervention team. Verbal instructions and movements were repeated as often as required, and participants were given additional time to process the instruction and initiate the movement. When a participant was unable to follow the proposed sequence, the movement was performed more slowly, and the sequence was simplified by reducing the number or complexity of its components. Physical or tactile guidance was provided when necessary to facilitate movement initiation, comprehension, and safe execution.

The programme was conducted in a spacious gymnasium equipped with well-maintained, shock-absorbing, non-slip flooring and immediate access to armrest chairs, facilitating rest breaks as needed. Each session was conducted by two physiotherapists specialised in motor exercise programming and two nurses experienced in performing Portuguese folk dance. An optimal group size of up to eight participants was observed for most sessions.

Exercise progression was individualised and based on the participant’s ability to perform the movement safely, maintain balance without requiring increased assistance, understand the instruction, complete the proposed sequence, and remain synchronised with the rhythm. When these criteria were met, progression involved increasing the number of repetitions and movement amplitude, combining simultaneous upper- and lower-limb movements, and introducing directional changes and rotation. When participants experienced difficulty completing the proposed exercise, the task was simplified by reducing movement speed and amplitude, removing rotational components, decreasing the number of movements in the sequence, or performing only the upper- or lower-limb components.

Perceived exertion was assessed using the Borg Rating of Perceived Exertion scale (Borg, 1998) at the end of each session and additionally during the session whenever the intervention team observed signs of increased exertion or reduced exercise tolerance.

A detailed outline of the training session structure is provided in Table 1.

**Table 1:** Structure of the training sessions.

Phase 1: warm-up (5 min)	Group warm-up: begin with gentle, full-body movements targeting the neck, shoulders, arms, trunk, hips, and knees. Perform these slowly and rhythmically to familiar music, with the instructor demonstrating and mirroring the actions to foster participation. Simple rhythmic steps: progress to basic steps like step-touch or gentle marching in place to gradually elevate heart rate and increase alertness. Seated option: for those preferring or needing seated activity, rhythmic marching or coordinated arm, and leg movements can be done while seated. Vocal activation: incorporate short vocal and breathing exercises—such as holding an “ahh”, gentle humming, and pitch glides—to support attention, breath control, and emotional engagement.
Phase 2: technical exercises (Two exercises per session) (10 min)	Two simple, repetitive movement exercises are performed in each session to enhance motor coordination and body awareness. Examples include: Trunk rotation. Step-touch sideways. Moving the hips (side-ways). Weight shifting. Step-touch forward-backwards. Basic step of the merengue. Sliding to the side. Twist.
Phase 3: directed improvisation (10 min)	Participants are encouraged to move freely in response to verbal or musical cues. The instructor offers simple directions such as, “move both arms up”, “stretch wide”, or “sway together”. When appropriate, participants take turns leading a movement for others to imitate, which promotes autonomy, memory recall, and social connection.
Phase 4: dance routine (10 min)	The instructor demonstrates the adapted Vira sequence, integrating the learned movements into a short, repetitive dance. In the first week, participants practise forming a circle, raising their arms, shifting weight from one leg to the other, taking two small steps forward, lightly tapping a foot, and then returning to the starting position. In weeks two and three, the sequence incorporates a slow 360° rotation before the forward steps. In the final stage, participants dance in a counterclockwise circle, keeping rhythm with the music and receiving assistance as needed.
Phase 5: relaxation (5 min)	The session concludes with slow, flowing movements set to soft music, incorporating gentle stretching, mindful breathing, and upper limb relaxation.

### 2.6 Data collection

Participant characteristics, including sex, age, history of dementia, and comorbidities, were extracted from the participants’ clinical records. Acceptability was predefined across four domains to inform programme refinement and a subsequent randomised controlled trial, as follows:

1. Enrolment—the number of eligible participants who have consented to and entered the programme.
2. Retention—the number of sessions attended as well as the percentage of enrolled participants who completed the final assessments.
3. Safety—monitored continuously during each intervention session by the two physiotherapists and two nurses delivering the programme. An adverse event was defined as any undesirable physical or behavioural occurrence during a session that required additional assistance, modification or interruption of the activity, clinical assessment, or discontinuation of participation. Monitored events included falls, near-falls, injuries, pain, dizziness, cardiovascular symptoms, excessive fatigue, agitation, refusal associated with distress, and other behavioural signs requiring modification or discontinuation of the session. Any adverse event identified was documented by the intervention team in the session record. Safety monitoring was limited to the intervention sessions.
4. Satisfaction—evaluated through a brief survey and semi-structured exit interviews.

The satisfaction questionnaire comprised five items assessing overall satisfaction with the intervention, perceived usefulness for balance, perceived difficulty, willingness to participate again, and willingness to recommend the programme to others. Given the participants’ cognitive impairment, the questionnaire was administered orally by a member of the research team. Questions and response options were read aloud, repeated or rephrased when necessary, and participants were given additional time to respond. Assistance was limited to supporting comprehension.

The interviews consisted of semi-structured sessions that explored participants’ perspectives regarding the acceptability of the intervention and their perceived usefulness. Furthermore, they identified barriers and facilitators that could influence participation. The interview guide addressed participants’ overall experience of the intervention, enjoyment, perceived physical and emotional effects, cultural and social relevance, perceived difficulty, sense of safety, barriers to participation, and willingness to participate again. Given the participants’ moderate-to-severe dementia, questions were brief, concrete, and focused on one topic at a time. The interviewer used simple language, allowed additional time for participants to process and answer each question, and repeated or rephrased questions when necessary. Neutral prompts were used to encourage participants to elaborate without suggesting particular responses. Example questions included: “How did you feel during the dance sessions?”, “What did you enjoy most?”, and “Was anything difficult or uncomfortable?” Both the satisfaction questionnaire and the interviews were completed directly by the participants; caregivers or proxies did not respond on their behalf.

Feasibility was assessed using standardised tools administered one week before the programme (T0) and one week after its completion (T1). Cognitive function was evaluated through the measurement of global cognitive status using the MoCA, a brief screening instrument (score range 0–30) that assesses attention, executive functions, memory, language, visuospatial skills, abstraction, and orientation. Higher scores indicate better cognitive performance. Balance and postural control were measured by evaluating balance recovery with the Mini-Balance Evaluation Systems Test (Mini-BESTest), a 14-item performance-based clinical scale divided into four domains: anticipatory postural control, reactive (compensatory) postural control, sensory orientation, and dynamic gait. Each item is rated from 0 to 2 (0 = lowest function; 2 = highest), resulting in a total score range of 0 to 28, with higher scores indicating improved balance and postural control. The Mini-BESTest was administered by two rehabilitation nurses who were external to the research team and were not involved in delivering the intervention. Assessments were conducted using the standardised Mini-BESTest instructions and scoring criteria under consistent conditions at baseline and post-intervention.

### 2.7 Data analysis

#### 2.7.1 Quantitative evaluation

All data collected at T0 and T1 were analysed using jamovi (The Jamovi Project, 2025), which relies on the R statistical environment (R Core Team, 2025). Descriptive statistics, including counts, means, standard deviations, medians, and ranges, as well as 95% confidence intervals, were computed. Each satisfaction item was rated on a 3-point scale, coded from 1 (most favourable response) to 3 (least favourable response). Responses were analysed separately for each item using frequencies and percentages; no composite satisfaction score was calculated. To compare MoCA and Mini-BEST scores between T0 and T1, paired-samples analyses were performed. Normality was evaluated via the Shapiro–Wilk test; when the assumption of normality was satisfied (*p* ≥ 0.05), paired *t*-tests were performed, and effect sizes (Cohen’s dz) were reported. In cases where normality was not met, Wilcoxon signed-rank tests were employed. Additionally, Bayesian paired-samples *t*-tests were conducted using the BayesFactor package (Morey et al., 2024), incorporating a Cauchy prior (scale = 0.707) as recommended by Rouder et al. (2009), representing a medium-effect prior frequently used in Bayesian hypothesis testing.

#### 2.7.2 Qualitative evaluation

Qualitative data were analysed using thematic analysis guided by the six-phase approach described by Braun and Clarke (2006): familiarisation with the data, generation of initial codes, development of candidate themes, review of themes, definition and naming of themes, and production of the final report. Following verbatim transcription, two researchers independently read the transcripts several times to become familiar with the data and coded them using QDA Miner Lite. Coding was primarily inductive and semantic, focusing on participants’ explicit accounts while remaining attentive to the domains addressed in the interview guide.

The two researchers subsequently compared their codes and grouped related codes into candidate themes representing recurring patterns across the dataset. Candidate themes were reviewed against both the coded extracts and the complete transcripts to assess their internal coherence, distinctiveness, and relevance to the study aims. Coding or thematic disagreements were first discussed between the two researchers. When consensus could not be reached, a third researcher reviewed the relevant transcript extracts and codes, and the final decision was reached through discussion. Themes were then refined, defined, and named, and representative quotations were selected to illustrate each theme.

## 3 Results

A total of 10 participants were initially enrolled in the programme (Figure 1). Of these, 8 participants completed the full 4-week intervention and final assessments, resulting in a retention rate of 80%. The observed attrition rate (20%) was primarily attributed to health-related issues and scheduling constraints typical of long-term care settings. Therefore, all statistical measures and tests were conducted considering solely the eight participants who completed the program.

Eight participants completed assessments at baseline (T0) and post-intervention (T1), with ages ranging from 77 to 97 years (mean = 85.9; SD = 7.41). The sample consisted predominantly of females (*n* = 7; 87.5%), with one male participant (12.5%). Overall, participants were in their mid-eighties, consistent with the typical age profile of residents in long-term care facilities. Baseline participant characteristics, including cognitive status, balance performance, and the level of support required during the intervention, are presented in Table 2. Of the eight participants who completed the intervention, three performed the dance activities with physical assistance, and one participated while seated in a wheelchair.

**Table 2:** Baseline characteristics and participation mode of participants who completed the intervention.

Characteristic	Value
Participants, *n*	8
Age, years, mean ± SD	85.9 ± 7.41
Age range, years	77–97
Female sex, *n* (%)	7 (87.5)
Male sex, *n* (%)	1 (12.5)
Documented clinical diagnosis of dementia, *n* (%)	8 (100)
Moderate-to-severe dementia, *n* (%)	8 (100)
Baseline MoCA, mean ± SD	9.13 ± 4.16
Baseline MoCA, median (range)	8.50 (4–16)
Baseline Mini-BESTest, mean ± SD	16.13 ± 4.61
Baseline Mini-BESTest, median (range)	17.00 (7–20)
Standing with physical assistance, *n* (%)	3 (37.5)
Seated in a wheelchair, *n* (%)	1 (12.5)

The average number of sessions attended by the participants was 6.88 (SD = 0.84) out of a total of 8 sessions, corresponding to an attendance rate of 85.94 ± 10.43% across the planned sessions (Table 3). Most participants (62.5%) missed no more than one session, demonstrating strong adherence and engagement with the intervention schedule.

**Table 3:** Attendance and adherence.

Variable	*n*	Mean	Median	SD	Minimum	Maximum
Attendance	8	6.88 (85.94%)	7.00 (87.50%)	0.835 (10.43%)	6 (75.0%)	8 (100%)

All participants who completed the programme underwent post-intervention assessments, resulting in a completion rate of 80% relative to the total number initially enrolled.

Satisfaction was evaluated using a brief structured questionnaire (3-point Likert scale) and semi-structured exit interviews. All participants (100%) reported high overall satisfaction, perceived usefulness for balance improvement, and willingness to repeat or recommend the activity.

No falls, near-falls, injuries, pain episodes, dizziness, cardiovascular symptoms, excessive fatigue, agitation, or behavioural incidents requiring exercise modification or session discontinuation were observed during any intervention session (0% incidence).

Descriptive statistics (Table 4) showed an increase in Mini-BESTest scores from T0 (*m* = 16.13, SD = 4.61) to T1 (*m* = 17.50, SD = 5.18). In contrast, MoCA scores remained stable, with an unchanged median of 8.50, the same range of 4–16, and similar variability at T0 (SD = 4.16) and T1 (SD = 3.99).

**Table 4:** Descriptive statistics for Mini-BESTest and MoCA.

Variable	Evaluation moment	*n*	Mean	Median	SD	Minimum	Maximum
Mini-BESTest	T0	8	16.13	17.00	4.61	7	20
	T1	8	17.50	19.50	5.18	7	22
MoCA	T0	8	9.13	8.50	4.16	4	16
	T1	8	9.25	8.50	3.99	4	16

Normality of the T1–T0 difference scores was assessed using the Shapiro–Wilk test, which indicated no significant departure from normality for Mini-BESTest difference scores (*w* = 0.906, *p* = 0.324). Therefore, a paired-sample Student’s *t*-test was used to compare pre- and post-intervention values for Mini-BESTest scores. The results showed a statistically significant increase from T0 to T1 (Mean Difference (T1–T0) = 1.38, SE = 0.324, *t* (7) = 4.25, *p* = 0.004, *d* = 1.5, 95% CI for *d* = [0.44, 2.51]). The Bayesian analysis yielded BF_10_ = 14.00 (numerical error ≈ 5.76 × 10^−7^), corresponding to BF_01_ = 0.071. Thus, the observed Mini-BESTest scores were 14 times more likely under the alternative hypothesis than under the null hypothesis, providing strong evidence for a change in Mini-BESTest scores.

For MoCA, the Shapiro–Wilk test indicated that the T1–T0 difference scores departed significantly from normality (*w* = 0.418, *p* < 0.001); therefore, a Wilcoxon signed-rank test was used. The test indicated no statistically significant difference between T0 and T1 (*w* = 1.00, *p* = 1.00). However, 7 out of 8 pairs were tied, substantially limiting the informativeness of the test. The Bayesian analysis yielded BF_10_ = 0.50 (numerical error ≈ 9.03 × 10^−5^), corresponding to BF_01_ = 2.00. Thus, under the specified Bayesian model, the observed data were twice as likely under the null hypothesis as under the alternative hypothesis, providing anecdotal evidence for no change in global cognitive function.

Balance scores showed a statistically significant pre-post increase, while cognitive performance remained stable, over the implementation period.

### 3.1 Interview data

Qualitative findings suggested that participants perceived the intervention as meaningful, enjoyable, and emotionally engaging. Three interrelated themes emerged from the interviews: (i) emotional and physical wellbeing, (ii) social and cultural connectedness, and (iii) perceived barriers to participation.

Participants frequently described the sessions as emotionally uplifting and stimulating. Several reported feeling more active, motivated, and physically confident following participation. Some also perceived improvements in movement fluidity and bodily comfort, particularly during standing, and walking activities. The intervention appeared to generate a sense of vitality and positive emotional engagement, as reflected in statements such as: *“I felt more cheerful afterwards”* and *“It helped both my body and my mind. I felt lighter”*. Others associated participation with increased ease of movement, noting that *“my movements became smoother, and I had less stiffness”*.

The social and culturally familiar nature of the intervention also emerged as a central facilitator of adherence. Participants highlighted enjoyment derived from group interaction, shared participation, and the familiarity of music and dance traditions. For several individuals, the intervention represented not only a physical activity but also a socially meaningful experience connected to identity, enjoyment, and interpersonal connection. This was reflected in comments such as: *“It was fun, and we were together, we talked and laughed”* and *“Because it involved dance, it made me want to move, the music helped”*. The supportive attitude of the staff was similarly identified as an important contributor to sustained engagement, with participants emphasising that *“the instructors were very kind and patient”*.

Despite the overall positive experience, some participants identified physical limitations that occasionally interfered with participation. The most commonly reported barriers included impaired balance, tremors during walking, and fatigue. These limitations were expressed in statements such as: *“My balance is not good”*, *“My legs shake when I start walking”*, and *“I get tired more easily on some days”*. Nevertheless, participants generally reported feeling safe and supported throughout the sessions, suggesting that the adaptations implemented during the intervention were appropriate for this population.

## 4 Discussion

This pilot study sought to evaluate the acceptability and feasibility of an adapted Portuguese folk dance–based intervention for older adults with moderate-to-severe dementia and balance impairment. The findings provide preliminary evidence that the intervention is both feasible and well accepted in a long-term care context, demonstrating promising engagement and safety outcomes.

Retention (80%) and attendance (85.9%) indicate that most participants were willing and able to engage in regular sessions over the 4 weeks. These figures align with feasibility benchmarks reported in early-phase intervention studies in long-term care, where health-related attrition and logistical barriers, such as staff availability, scheduling constraints, and fluctuations in residents’ clinical status, are frequently observed, particularly among people living with dementia (Lam et al., 2018a; Mody et al., 2008).

Importantly, our results are comparable to retention rates reported in the systematic review by Di Lorito et al. (2020), which found adherence rates between 16 and 100% (with a mean adherence of 70%, SD = 21) among individuals with mild cognitive impairment or mild dementia participating in exercise interventions. Despite involving individuals with moderate-to-severe dementia (mean MoCA = 9.13 ± 4.16), participants achieved a comparable level of engagement. This finding may suggest a potential for implementing structured and meaningful physical activities even among those with more advanced cognitive impairment, provided these are appropriately adapted to individual cognitive and physical capacities.

Acceptability was high across all indicators. Quantitative findings revealed unanimous satisfaction, perceived usefulness for improving balance, and strong willingness to repeat or recommend the programme. These outcomes underscore that the intervention was not only tolerated but positively received, with participants expressing enjoyment and motivation. The qualitative data reinforced these results, revealing the programme’s social, emotional, and cultural relevance. Participants described the sessions as fun, stimulating, and meaningful, emphasising the music, rhythm, and group interaction as central motivators. Similar findings have been reported in studies exploring dance and music-based therapies for people with dementia, where cultural familiarity and emotional resonance enhance engagement and satisfaction (Mabire et al., 2019).

From an outcomes perspective, a statistically significant pre–post increase in Mini-BESTest scores was observed. However, the dance programme was delivered as an adjunct to usual care, which included ongoing occupational and physical therapy. These concurrent rehabilitation activities may have contributed to changes in balance and mobility and may also have influenced participants’ functional capacity, exercise tolerance, and ability to engage safely in the intervention. Because the study did not include a usual-care control group, the independent contribution of the dance programme cannot be determined. The observed balance change should therefore be interpreted as an exploratory signal rather than as an effect attributable specifically to the Portuguese folk dance intervention.

These results are nevertheless directionally consistent with prior evidence suggesting that dance-based or rhythm-guided movement may enhance postural stability and functional mobility in people with cognitive impairment (Ruiz-Muelle and López-Rodríguez, 2019; Tao et al., 2023). It is also important to consider the limited exposure in this pilot, a short-duration programme intentionally designed to prevent fatigue and cognitive saturation, in accordance with international recommendations for exercise and activity-based interventions in dementia. Authors emphasise the importance of brief, structured, and enjoyable sessions to maintain engagement, safety, and adherence (Lam et al., 2018a; Di Lorito et al., 2020). While this design choice likely supported participation and tolerability, it may also have constrained the magnitude of measurable functional change.

The implementation findings are particularly relevant given the functional heterogeneity of the participants. Of the eight participants who completed the programme, three performed the dance with physical assistance, and one participated while seated in a wheelchair. Thus, half of the participants completed the intervention using an adapted mode of participation within the same group sessions. This indicates that the protocol could accommodate different levels of balance and mobility impairment when close supervision, individual cueing, physical assistance, and seated adaptations were available. The absence of adverse events under these conditions is encouraging, although safety cannot be assumed in settings with different staffing, rehabilitation provision, or environmental resources.

Feasibility was demonstrated under specific staffing and environmental conditions. However, the four-professional delivery model is resource intensive and may not be reproducible in long-term care facilities with fewer available staff.

Implementation additionally required a sufficiently large space for group movement and wheelchair manoeuvring, shock-absorbing non-slip flooring, chairs with armrests, and access to gait belts when physical assistance was required. Although specialised technological equipment was not needed, staff time, professional training, suitable space, and safety equipment represent important resource requirements. Resource use and costs were not formally evaluated in this pilot study; therefore, no conclusions can be drawn regarding affordability or cost-effectiveness. Future studies should prospectively assess staff time, training requirements, space and equipment needs, and the costs associated with different delivery models.

The cultural adaptation of the intervention through the Portuguese folk dance Vira was perceived by participants as socially and personally meaningful and may have contributed to their enjoyment and motivation. However, because all participants were familiar with the Vira and were already receptive to participating, the present design cannot establish whether cultural familiarity was itself responsible for engagement or acceptability. Future studies should record prior familiarity and interest as participant characteristics and, where feasible, recruit people with varying degrees of familiarity with Portuguese folk dance.

### 4.1 Limitations

This study has several limitations that should be acknowledged. First, as noted earlier, the small sample size and single-site design limit statistical precision; consequently, the findings should be interpreted as exploratory and indicative rather than confirmatory. Second, recruitment proceeded from a public announcement describing a Portuguese folk dance programme, and eligibility required familiarity with the Vira and willingness to take part. Although no residents were excluded on this basis, selection nonetheless operated through self-registration, since residents indifferent or averse to this form of activity may have been less likely to register. The satisfaction and adherence figures reported here should therefore be read as applying to residents already receptive to the activity rather than to the resident population. Because all participants were familiar with the Vira, the present design cannot determine whether familiarity itself contributed to engagement and acceptability. Future studies should recruit participants irrespective of prior familiarity and record familiarity and interest as participant characteristics. Third, the short intervention period—although deliberately designed to minimise participant fatigue and support adherence—may have constrained the magnitude of functional gains. Fourth, because assessments relied in part on self-reported and observational data, response bias cannot be ruled out, and participants’ cognitive impairment may have further influenced reporting and performance. Self-reported satisfaction should be interpreted particularly cautiously, as moderate-to-severe cognitive impairment may have affected participants’ comprehension, recall, and response consistency. In addition, oral administration of the questionnaire by a member of the research team may have increased the risk of acquiescence or social desirability bias. These considerations compound the selection described above: the reported satisfaction should not be read as an estimate of how the intervention would be received by residents without a prior connection to Portuguese folk dance. Finally, the intervention was delivered by four healthcare professionals to a group of up to eight participants. Although this staffing configuration enabled close supervision, individualised assistance, and adaptation to different levels of balance and cognitive impairment, it may be difficult to reproduce in long-term care settings with fewer available professionals. The feasibility and scalability of alternative delivery models therefore remain to be established, including whether the intervention can be implemented safely with fewer professionals or through the training of existing long-term care staff. Despite these limitations, the study offers preliminary evidence on the feasibility of delivering a culturally adapted, dance-based intervention to residents with moderate-to-severe dementia, including those requiring physical assistance or wheelchair-based participation, and on its acceptability among residents receptive to this form of activity.

## 5 Conclusion

Taken together, the results provide preliminary evidence supporting the feasibility, safety, and acceptability of the adapted Portuguese folk dance–based programme. High satisfaction and adherence, together with the absence of adverse events, indicate that the intervention was deliverable and well tolerated under the staffing and environmental conditions used in this study. The observed pre-post change in balance scores represents a preliminary, exploratory signal warranting confirmation in a controlled trial. This study suggests that a culturally grounded, group-based dance intervention can be safely implemented and is perceived as meaningful and enjoyable by older adults with dementia. Future studies should retain its person-centred and culturally embedded nature while addressing scalability and staff training requirements for broader application.

## Data Availability

The raw data supporting the conclusions of this article will be made available by the authors, without undue reservation.

## References

[ref1] Aguiñaga S. Marquez D. X. (2017). Feasibility of a Latin dance program for older Latinos with mild cognitive impairment. Am. J. Alzheimers Dis. Other Dement. 32, 479–488. doi: 10.1177/1533317517719500, 28683560 PMC10852848

[ref2] Andrade-Guerrero J. Martínez-Orozco H. Villegas-Rojas M. M. Santiago-Balmaseda A. Delgado-Minjares K. M. Pérez-Segura I. . (2024). Alzheimer’s disease: understanding motor impairments. Brain Sci. 14:1054. doi: 10.3390/brainsci14111054, 39595817 PMC11592238

[ref3] Asnaani A. Hofmann S. G. (2012). Collaboration in multicultural therapy: establishing a strong therapeutic alliance across cultural lines. J. Clin. Psychol. 68, 187–197. doi: 10.1002/jclp.21829, 23616299 PMC3641707

[ref4] Baird A. Samson S. (2015). Music and dementia. Prog. Brain Res. 217, 207–235. doi: 10.1016/bs.pbr.2014.11.028, 25725917

[ref5] Bajwa R. K. Goldberg S. E. Van der Wardt V. Burgon C. Di Lorito C. Godfrey M. . (2019). A randomised controlled trial of an exercise intervention promoting activity, independence and stability in older adults with mild cognitive impairment and early dementia (PrAISED)—a protocol. Trials 20:815. doi: 10.1186/s13063-019-3871-9, 31888709 PMC6937783

[ref6] Borg G. (1998). Borg’s Perceived Exertion and Pain Scales. Champaign: Human Kinetics.

[ref7] Bracco L. Pinto-Carral A. Hillaert L. Mourey F. (2023). Tango-therapy vs physical exercise in older people with dementia; a randomised controlled trial. BMC Geriatr. 23:693. doi: 10.1186/s12877-023-04342-x 37875856 PMC10598907

[ref8] Braun V. Clarke V. (2006). Using thematic analysis in psychology. Qual. Res. Psychol. 3, 77–101. doi: 10.1191/1478088706qp063oa

[ref9] Conn V. S. Chan K. Banks J. Ruppar T. M. Scharff J. (2013). Cultural relevance of physical activity intervention research with underrepresented populations. Int. Q. Community Health Educ. 34, 391–414. doi: 10.2190/IQ.34.4.g, 25228486 PMC4388245

[ref10] Di Lorito C. Bosco A. Booth V. Goldberg S. Harwood R. H. Van der Wardt V. (2020). Adherence to exercise interventions in older people with mild cognitive impairment and dementia: a systematic review and meta-analysis. Prev. Med. Rep. 19:101139. doi: 10.1016/j.pmedr.2020.101139, 32793408 PMC7414005

[ref11] Fernandes J. B. Domingos J. Família C. Veríssimo J. Castanheira P. Menezes C. . (2023). Adapted Portuguese folk dance intervention for subacute rehabilitation post-stroke: study protocol. Front. Public Health 11:1200093. doi: 10.3389/fpubh.2023.1200093, 37663853 PMC10470121

[ref12] Fonseca I. Rueda M. Cabanzo C. (2025). The effect of dance interventions on wellbeing dimensions in older adults: a systematic review. Front. Sports Act. Living 7:1594754. doi: 10.3389/fspor.2025.1594754, 40787603 PMC12331657

[ref13] Fujita K. Sugimoto T. Noma H. Kuroda Y. Matsumoto N. Uchida K. . (2024). Postural control characteristics in Alzheimer’s disease, dementia with Lewy bodies, and vascular dementia. J. Gerontol. A Biol. Sci. Med. Sci. 79:glae061. doi: 10.1093/gerona/glae061, 38412449 PMC10949438

[ref14] Jayakody S. Arambepola C. (2022). Determinants of quality of life among people with dementia: evidence from a south Asian population. BMC Geriatr. 22:745. doi: 10.1186/s12877-022-03443-3, 36096721 PMC9469587

[ref15] Kim S. J. Kim H. D. (2024). Relationship between falls, cognitive decline, and dementia in older adults: insights from the Korean longitudinal study of aging, 2006–2020. Exp. Gerontol. 194:112481. doi: 10.1016/j.exger.2024.112481, 38871235

[ref16] Lam H. R. Chow S. Taylor K. Chow R. Lam H. Bonin K. . (2018b). Challenges of conducting research in long-term care facilities: a systematic review. BMC Geriatr. 18:242. doi: 10.1186/s12877-018-0934-9, 30314472 PMC6186062

[ref17] Lam F. M. Huang M. Z. Liao L. R. Chung R. C. Kwok T. C. Pang M. Y. (2018a). Physical exercise improves strength, balance, mobility, and endurance in people with cognitive impairment and dementia: a systematic review. J. Physiother. 64, 4–15. doi: 10.1016/j.jphys.2017.12.001, 29289581

[ref18] Levinger P. Goh A. M. Y. Dunn J. Katite J. Paudel R. Onofrio A. . (2023). Exercise intervention outdoor project in the community—results from the ENJOY program for independence in dementia: a feasibility pilot randomised controlled trial. BMC Geriatr. 23:426. doi: 10.1186/s12877-023-04132-5, 37438710 PMC10337151

[ref19] Long A. Robinson K. Goldberg S. Gordon A. L. (2019). Effectiveness of exercise interventions for adults over 65 with moderate-to-severe dementia in community settings: a systematic review. Eur. Geriatr. Med. 10, 843–852. doi: 10.1007/s41999-019-00236-7, 34652766

[ref20] Mabire J. B. Aquino J. P. Charras K. (2019). Dance interventions for people with dementia: systematic review and practice recommendations. Int. Psychogeriatr. 31, 977–987. doi: 10.1017/S1041610218001552, 30296957

[ref21] Manji I. Wells S. Dal Bello-Haas V. Fallavollita P. (2024). Impact of dance interventions on the symptoms of dementia: a mixed-methods systematic review. Arts Health 16, 64–88. doi: 10.1080/17533015.2023.2242390, 37559369

[ref22] Menezes A. C. Drumond G. Shigaeff N. (2022). Dance therapy and cognitive impairment in older people: a review of clinical data. Dement. Neuropsychol. 16, 373–383. doi: 10.1590/1980-5764-DN-2021-0103, 36530760 PMC9745973

[ref23] Mody L. Miller D. K. McGloin J. M. Freeman M. Marcantonio E. R. Magaziner J. . (2008). Recruitment and retention of older adults in aging research. J. Am. Geriatr. Soc. 56, 2340–2348. doi: 10.1111/j.1532-5415.2008.02015.x, 19093934 PMC2695395

[ref24] Montero-Odasso M. van der Velde N. Martin F. C. Petrovic M. Tan M. P. Ryg J. . (2022). World guidelines for falls prevention and management for older adults: a global initiative. Age Ageing 51:afac205. doi: 10.1093/ageing/afac205, 36178003 PMC9523684

[ref25] Morey R. D. Rouder J. N. Jamil T. Urbanek S. (2024) BayesFactor: Computation of Bayes Factors for Common Designs (R package version 0.9.12-4.6) [Computer software]. Comprehensive R Archive Network. Available online at: https://cran.r-project.org/web/packages/BayesFactor/BayesFactor.pdf (Accessed March 11, 2026).

[ref26] National Institute for Health and Care Care Excellence (2018). Dementia: Assessment, Management and Support for People Living with Dementia and their Carers. London: National Institute for Health and Care Excellence. 30011160

[ref27] R Core Team. (2025) R: A Language and Environment for Statistical Computing. R Foundation for Statistical Computing. Available online at: https://www.r-project.org/ (Accessed March 11, 2026).

[ref28] Ribas T. (1983). Danças Populares Portuguesas. Lisboa: Instituto de Cultura e Língua Portuguesa.

[ref29] Ribeiro S. Palhares P. Portugal M. (2020). Ethnomathematical study on folk dances: focusing on the choreography. Revemop 2, 1–16. doi: 10.33532/revemop.e202014

[ref30] Rouder J. N. Speckman P. L. Sun D. Morey R. D. (2009). Bayesian t tests for accepting and rejecting the null hypothesis. Psychon. Bull. Rev. 16, 225–237. doi: 10.3758/PBR.16.2.225, 19293088

[ref31] Ruiz-Muelle A. López-Rodríguez M. M. (2019). Dance for people with Alzheimer’s disease: a systematic review. Curr. Alzheimer Res. 16, 919–933. doi: 10.2174/1567205016666190725151614, 31345149

[ref32] Smebye K. L. Kirkevold M. (2013). The influence of relationships on personhood in dementia care: a qualitative, hermeneutic study. BMC Nurs. 12:29. doi: 10.1186/1472-6955-12-29, 24359589 PMC3878215

[ref33] Soysal P. Tan S. G. Smith L. (2021). A comparison of the prevalence of fear of falling between older patients with Lewy body dementia, Alzheimer’s disease, and without dementia. Exp. Gerontol. 146:111248. doi: 10.1016/j.exger.2021.111248, 33486068

[ref34] Tao D. Awan-Scully R. Ash G. I. Pei Z. Gu Y. Gao Y. . (2023). The effectiveness of dance movement interventions for older adults with mild cognitive impairment, Alzheimer’s disease, and dementia: a systematic scoping review and meta-analysis. Ageing Res. Rev. 92:102120. doi: 10.1016/j.arr.2023.102120, 37944706 PMC11262040

[ref35] The Jamovi Project Jamovi (version 2.6) [Computer Software] (2025). Available online at: https://www.jamovi.org (Accessed March 11, 2026).

[ref36] United Nations Department of Economic and Social Affairs, Population Division (2023). World Population Ageing 2023: Challenges and Opportunities of Population Ageing in the Least Developed Countries. New York, United Nations.

[ref37] Veronese N. Soysal P. Demurtas J. Solmi M. Bruyère O. Christodoulou N. . (2023). Physical activity and exercise for the prevention and management of mild cognitive impairment and dementia: a collaborative international guideline. Eur. Geriatr. Med. 14, 925–952. doi: 10.1007/s41999-023-00858-y 37768499 PMC10587099

[ref38] Wang S. Jiang Y. Yang A. Meng F. Zhang J. (2024). The expanding burden of neurodegenerative diseases: an unmet medical and social need. Aging Dis. 16, 2937–2952. doi: 10.14336/AD.2024.1071, 39571158 PMC12339136

[ref39] World Health Organisation (2025a) Ageing: Global Population. Geneva. Available online at: https://www.who.int/news-room/questions-and-answers/item/population-ageing (Accessed March 18, 2026).

[ref40] World Health Organisation (2025b) Dementia. Geneva. Available online at: https://www.who.int/news-room/fact-sheets/detail/dementia (Accessed March 18, 2026).

